# Supplementary material for: Mortality Prediction Model before Surgery for Acute Mesenteric Infarction: A Population-Based Study
Source: J Clin Med. 2022 Oct 8;11(19):5937. doi: 10.3390/jcm11195937 (PMC9571294; doi:10.3390/jcm11195937)
Supplement: Supplementary file 1 [file jcm-11-05937-s001.zip › jcm-1917487-supplementary.pdf]

Table S1. Translation of Comorbidities into ICD-9-CM Codes.

Diagnostic Category	ICD-9-CM Codes	Description
Myocardial infarction	410-410.9	Acute myocardial infarction
	412	Old myocardial infarction
Congestive heart failure	428-428.9	Heart failure
Vascular disease	443.9	Peripheral vascular disease, including intermittent claudication
	441-441.9	Aortic aneurysm
	785.4	Gangrene
	V43.4	Blood vessel replaced by prosthesis
	Procedure 38.48	Resection and replacement of lower limb arteries
Cerebrovascular disease	430-438	Cerebrovascular disease
Dementia (From RICPD and DD)	290-290.9	Senile and presenile dementias
Chronic pulmonary disease	490-496	Chronic obstructive pulmonary disease
	500-505	Pneumoconioses
	506.4	Chronic respiratory conditions due to fumes and vapors
Rheumatologic disease (From RICPD and DD)	710.0	Systemic lupus erythematosus
	710.1	Systemic sclerosis
	710.4	polymyositis
	714.0-714.2	Adult rheumatoid arthritis
	714.81	Rheumatoid lung
	725	polymyalgiarheumatica
Peptic ulcer disease	531-534.9	Gastric, duodenal and gastrojejunal ulcers
	531.4-531.7	Chronic forms of peptic ulcer disease (subset of above listing)
	532.4-532.7	
	533.4-533.7	
	534.4-534.7	
Severe liver disease (From RICPD and DD)	572.2-572.8	Hepatic coma, portal hypertension, other sequelae of chronic liver disease
	456.0-456.2	Esophageal varices
Diabetes	250-250.3	Diabetes with or without acute metabolic disturbances
	250.7	Diabetes with peripheral circulatory disorders
	250.4-250.6	Diabetes with renal, ophthalmic, or neurological manifestations
Hemiplegia or paraplegia	344.1	Paraplegia
	342-342.9	Hemiplegia
Dialysis (From RICPD)	403,404	Hypertensive chronic kidney disease
	585	Chronic renal failure
Any malignancy, including leukemia and lymphoma (From RICPD and DD)	140-172.9	Malignant neoplasms
	174-195.8	Malignant neoplasms
	200-208.9	Leukemia and lymphoma
	196-199.1	Secondary malignant neoplasm of lymph nodes and other organs

Abbreviations: RICPD: Registry of Catastrophic Illness Patient Database; DD: inpatient expenditures by admission.

Table S2. Logistic regression analysis of patient parameters potentially associated with 30-day mortality

	**Univariant analysis**		**Multivariable analysis**				
	OR	p	Adjusted OR	95% CI	p	B	Score
Age ≥62 years	2.614	<0.001	2.275	1.808-2.863	<0.001	0.822	**3**
Men	0.992	0.920					
Myocardial infarction	1.856	<0.001	1.227	0.893–1.686	0.206		
Congestive heart failure	2.159	<0.001	1.475	1.197–1.818	**<0.001**	0.389	1
Perivascular disease	1.188	0.046	1.077	0.904–1.284	0.404		
Cerebral disease	1.780	<0.001	1.275	1.041–1.562	**0.019**	0.243	1
Dementia	1.820	0.018	1.349	0.803–2.266	0.259		
Chronic obstructive pulmonary disease	1.609	<0.001	1.045	0.839–1.300	0.696		
Rheumatic disease	1.624	0.097	1.687	0.930–3.061	0.085		
Peptic ulcer disease	2.000	<0.001	1.513	1.259–1.819	**<0.001**	0.414	1
Severe liver disease	2.815	<0.001	3.140	1.988–4.960	**<0.001**	1.144	4
Hemiplegia	1.524	0.030	1.052	0.697–1.589	0.809		
Hemodialysis	2.208	<0.001	1.736	1.330–2.264	**<0.001**	0.551	2
Hypertension	1.755	<0.001	0.960	0.782–1.179	0.699		
Hyperlipidemia	1.249	0.090	0.777	0.587–1.028	0.078		
Gout	1.794	<0.001	1.231	0.932–1.626	0.143		
Malignancy	1.236	0.046	1.184	0.954–1.471	0.125		
Obese	0.000	0.999					
Diabetes	1.785	<0.001	1.307	1.080–1.580	**0.006**	0.267	1
Heart conduct disease	1.651	<0.001	1.152	0.933–1.422	0.188		
Laparoscope operation	0.409	0.796					

OR, odds ratio; CI, confidence interval.

Table S3. The different scoring systems.

Factors	B	Multiplied Number								
		1	1.3	1.5	1.9	2.1	2.2	2.8	3.1	3.7
Age > 62 y/o	0.822	1	1	1	2	2	2	2	3	3
Congestive heart failure	0.389	0	1	1	1	1	1	1	1	1
Cerebral disease	0.243	0	0	0	0	1	1	1	1	1
Peptic ulcer disease	0.414	0	1	1	1	1	1	1	1	2
Severe liver disease	1.144	1	1	2	2	2	3	3	4	4
Hemodialysis	0.551	1	1	1	1	1	1	2	2	2
Diabetes	0.267	0	0	0	1	1	1	1	1	1
Derivation cohort Result	#**AUC of ROC**	0.623	0.661	0.664	0.669	0.672	0.673	0.676	0.677	0.675
	*Calibration $\chi 2$	8.207	22.335	21.844	26.426	28.719	26.472	26.718	19.887	23.110
	Calibration p	0.004	<0.001	<0.001	<0.001	<0.001	<0.001	<0.001	0.001	<0.001

* Calibration was done with Hosmer–Lemeshow goodness-of-fit test # all p<0.05.

Table S4. Distribution of patients according to the acute mesenteric ischemia mortality score.

Score	Derivation cohort			Validation Cohort			Total Cohort		
	Total number	30-Days Mortality	Mortality rate	Total number	30-Days Mortality	Mortality rate	Total number	30-Days Mortality	Mortality rate
0	791	35	4.4%	201	7	3.5%	992	42	4.2%
1	207	27	13.0%	62	3	4.8%	269	30	11.2%
2	77	8	10.4%	19	3	15.8%	96	11	11.5%
3	943	129	13.7%	236	35	14.8%	1179	164	13.9%
4	859	183	21.3%	195	40	20.5%	1054	223	21.2%
5	530	143	27.0%	125	34	27.2%	655	177	27.0%
6	265	79	29.8%	73	23	31.5%	338	102	30.2%
7	132	47	35.6%	36	14	38.9%	168	61	36.3%
8	77	19	24.7%	16	7	43.8%	93	26	28.0%
9	27	10	37.0%	11	1	9.1%	38	11	28.9%
10	5	3	60.0%	3	0	0.0%	8	3	37.5%
11	5	1	20.0%	1	1	100.0%	6	2	33.3%
12	0	0	N.A.	0	0	N.A.	0	0	N.A.
13	0	0	N.A.	1	0	0.0%	1	0	0.0%

Scores: 0, very low risk; 1–3, low risk; 4-6, intermediate risk; 7–13, high risk.

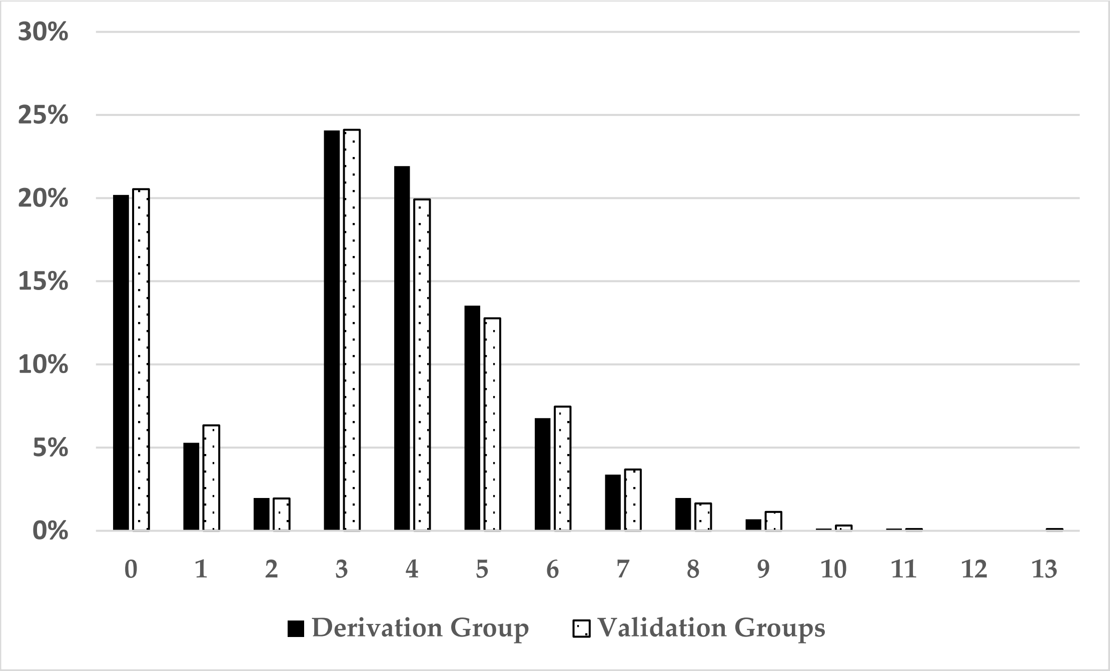

Figure S1: Distribution of Score in two groups
